# Assessment of Risk Factors for *Cryptosporidium* Infection in Hospitalized Patients from Romania

**DOI:** 10.3390/jcm14134481

**Published:** 2025-06-24

**Authors:** Rodica Georgiana Dărăbuș, Marius Stelian Ilie, Diana Maria Darabuș, Voichița Lăzureanu, Ovidiu Roșca, Tudor Rareș Olariu

**Affiliations:** 1 Discipline of Parasitology, Department of Infectious Diseases, Victor Babes University of Medicine and Pharmacy, 300041 Timisoara, Romania; georgiana.darabus@umft.ro (R.G.D.); rolariu@umft.ro (T.R.O.); 2 Center for Diagnosis and Study of Parasitic Diseases, Department of Infectious Disease, Victor Babes University of Medicine and Pharmacy, 300041 Timisoara, Romania; 3 Discipline of Parasitology, Faculty of Veterinary Medicine, University of Life Sciences “King Mihai I”, 300645 Timisoara, Romania; mariusilie@usvt.ro; 4 Oftalmo Sensory-Tumor Research Center—ORL (EYE-ENT), Ophthalmology Department, “Victor Babes” University of Medicine and Pharmacy, 300041 Timisoara, Romania; 5 Infectious Diseases Clinic, Victor Babes University of Medicine and Pharmacy, 300041 Timisoara, Romania; lazureanu.voichita@umft.ro; 6 Methodological and Infectious Diseases Research Center, Department of Infectious Diseases, “Victor Babes” University of Medicine and Pharmacy, 300041 Timisoara, Romania; ovidiu.rosca@umft.ro

**Keywords:** *Cryptosporidium*, Romania, human prevalence, risk factors

## Abstract

**Background/Objectives**: This study aimed to identify and analyze the risk factors associated with Cryptosporidium infection in hospitalized patients in western Romania. **Methods:** A total of 312 patients, aged between 2 months and 90 years and residing in both urban and rural communities, were included. Stool samples were collected and analyzed using the CerTest Crypto qualitative chromatographic test and the modified Ziehl–Neelsen staining method (Henricksen & Pohlenz). Risk factors were assessed through a questionnaire completed by patients or by the parents of pediatric patients. **Results:** The overall prevalence of Cryptosporidium infection was 5.77%. Among the evaluated risk factors, only the area of residence showed a statistically significant association (*p* < 0.05), with a higher prevalence in urban areas (9.2%) compared to rural areas (3.6%). Other factors—including age, gender, contact with animals, pet ownership, handwashing after animal contact, type of housing, fruit washing habits, use of potable water, use of public transportation, international travel, and visits to playgrounds or swimming pools—were not significantly associated with infection. **Conclusions:** These findings suggest that urban residency may be a significant factor in Cryptosporidium transmission and may inform future research and the development of targeted public health strategies.

## 1. Introduction

*Cryptosporidium* is a protozoan parasite primarily affecting the digestive system of various vertebrate species. Since its first identification in humans in 1976, *Cryptosporidium* is considered across the globe to be a significant cause of gastrointestinal disorders [1,2]. In immunocompetent individuals, a *Cryptosporidium* infection is usually self-limiting. However, in immunocompromised patients, it can be severe, progressive, and persistent [3,4]. Symptoms in these patients typically include profuse watery diarrhea, abdominal pain, mild fever, sensations of nausea, episodes of vomiting, and unintended weight reduction [5].

Approximately 20 species of *Cryptosporidium* are known to infect humans, with *C. hominis*, *C. parvum*, *C. meleagridis*, *C. canis*, and *C. felis* representing the species most frequently identified [6,7]. Between 2015 and 2017, a study in France identified 210 cases of cryptosporidiosis in immunodeficient patients, with *C. parvum* and *C. hominis* being the predominant species. The infection was most commonly associated with organ transplantation (49%) and HIV infection (30%) [4]. Person-to-person transmission of *C. hominis* within households is considered significant and, thus, it is recommended that *Cryptosporidium* testing be included in routine investigations of gastrointestinal pathogens [8].

An analysis across countries with different levels of development revealed that in high-income countries, cryptosporidiosis affects individuals regardless of age or immune status. In contrast, the infection shows higher prevalence among pediatric populations and HIV-positive individuals in low- and middle-income nations. In high-income countries, cryptosporidiosis is most often diagnosed in children over the age of two, while in low- and middle-income regions, it tends to affect children under two years of age [9]. The same study indicated that risk factors in high-income countries are linked to international travel, contact with infected individuals or animals, and swimming. In contrast, in low-income settings, risk factors are more related to poor hygiene, overcrowding, and household diarrhea cases.

Socio-economic factors, acute and chronic malnutrition, inadequate healthcare access, low maternal education levels, and the use of public tap water have been associated with both cryptosporidiosis and non-cryptosporidial diarrhea [10]. Additionally, the prevalence of the *Cryptosporidium* infection is significantly higher in urban environments, likely due to higher population density, inadequate sanitation, and associated comorbidities [11,12,13,14]. Certain research findings suggest an association between cryptosporidiosis and the use of stored drinking water, as well as exposure to animals like cattle, cats, and rodents [15]. Other risk factors reported in the literature include exposure to agricultural environments, particularly domestic animal feces and manure [14].

Human cryptosporidiosis remains insufficiently documented in Romania. Although several studies have addressed this topic, to the best of our knowledge, none have provided a comprehensive evaluation of the risk factors associated with *Cryptosporidium* infection. The present study aims to identify and assess potential risk factors for the *Cryptosporidium* infection among hospitalized patients in Romania.

## 2. Materials and Methods

### 2.1. Samples and Data Collection

A total of 312 fresh fecal specimens were collected in sterile stool containers during 2024 and transported to the lab for testing. The samples were obtained from patients hospitalized at the Victor Babeș Infectious Diseases Hospital in Timișoara, western Romania. The study included all patients who were hospitalized from February to April 2024. The exclusion criterion was solely based on patient refusal. As there were no patients who refused to participate in the study, all patients from this specified period were interviewed and examined. Every patient enrolled in the study was administered treatment targeting the primary illness that led to their hospitalization.

### 2.2. Study Participants

The research participants comprised male and female patients aged between 2 months and 90 years. The participants were divided into the seven following age brackets: under 1 year, 1 to 5 years, 6 to 10 years, 11 to 17 years, 18 to 39 years, 40 to 59 years, and 60 years or older. Hospitalized individuals resided in a mix of urban and rural areas within western Romania. At the time of sample collection, some of the participants presented with digestive symptoms while others did not. Their exposure to animals varied, from having direct ownership of animals, to incidental contact with various animal species not owned by them.

### 2.3. Laboratory Tests

Human cryptosporidiosis was identified through a combination of rapid testing methods and the modified Ziehl–Neelsen stain described by Henriksen and Pohlenz [16,17].

#### 2.3.1. CerTest Crypto

The CerTest Crypto is a qualitative chromatographic assay intended to identify *Cryptosporidium* oocysts in fecal samples. It demonstrates sensitivity and specificity exceeding 99% (CerTest BIOTEC S.L., San Mateo de Gállego, Zaragoza, Spain). The test was conducted following the manufacturer’s guidelines [16].

#### 2.3.2. Modified Ziehl–Neelsen Staining (Henriksen & Pohlenz Method)

The modified Ziehl–Neelsen staining procedure, based on the method established by Henriksen and Pohlenz in 1981, was conducted on all 312 fresh stool samples tested with CerTest Crypto. This method is widely accepted as the reference standard for *Cryptosporidium* detection. Under 100× magnification with immersion oil, *Cryptosporidium* oocysts appeared as round or oval, with red-stained bodies set against a greenish-blue background. Other elements such as cells, bacteria, and yeasts stained green, making it easy to distinguish *Cryptosporidium* oocysts from surrounding debris [17].

### 2.4. Risk Factors

To evaluate possible risk factors, the participants, or the parents or guardians of child patients, filled out a detailed questionnaire. The questionnaire gathered data regarding the area of residence, gender, presence of digestive symptoms, pet ownership, animal contact, handwashing practices after animal contact, consumption of potable water, washing of fruits and vegetables before consumption, frequency of swimming pool and playground visits, use of public transportation, frequency of international travel, and awareness of cryptosporidiosis.

The questionnaire was developed by a multidisciplinary team, including epidemiologists, physicians, veterinarians, and infectious disease experts. Moreover, the questionnaire was developed following established protocols and guidelines issued by the European Centre for Disease Prevention and Control (ECDC) concerning cryptosporidiosis. This approach guaranteed that the questionnaire remained relevant and aligned with international standards for assessing *Cryptosporidium* risk factors [18].

### 2.5. Statistical Analysis

Statistical evaluations were conducted using Epi Info version 7.2.6 (Centers for Disease Control and Prevention, Atlanta, GA, USA, 2023), IBM SPSS Statistics version 20.0 (IBM Corp., Armonk, NY, USA), and the statsmodels library version 0.14.0 in Python (Python Software Foundation, Wilmington, DE, USA). The Fisher–Mantel–Haenszel exact test (two-tailed) was used to assess the significance of risk factors, with statistical significance defined as a *p*-value ≤ 0.05. Logistic regression analysis was performed using validated statistical methods, and the authors reviewed and verified the results and their interpretation.

### 2.6. Ethical Approval

Approval for the study was granted by the Ethics Committee of Victor Babeș University of Medicine and Pharmacy, Timișoara (Approval No. 12/17 March 2023). Prior to enrollment, written informed consent was obtained from all participants or, in the case of minors, from their parents or legal guardians.

## 3. Results

A complete concordance was observed between the CerTest Crypto assay and Ziehl–Neelsen staining, as all positive samples were confirmed by both methods.

### 3.1. Age and Gender

*Cryptosporidium* was identified across all age groups, with the exception of those aged 11–17 years. This may be due to the low number of hospitalized cases in this category (Table 1).

The highest prevalence was observed in the 1–5-year age group (7.8%). However, no significant differences in prevalence were found among age groups. The youngest age at which *Cryptosporidium* was detected was 10 months; the protozoan was identified in a male infant from a rural area who lived in a house and had diarrhea at the time of sample collection. The oldest patient with a confirmed infection was an 81-year-old woman from an urban area who lived in an apartment and also had diarrhea. No significant differences in *Cryptosporidium* prevalence were observed between genders.

### 3.2. Environmental and Lifestyle Risk Factors

A significantly higher infection rate was observed among participants from urban areas (9.2%) than those from rural regions (3.6%) (*p* < 0.05). In an univariate statistical analysis, the adjusted Mantel–Haenszel test and maximum likelihood adjusted (MLE) estimates of the odds ratio for rural versus urban were 0.469 (0.239–0.921) and 0.476 (0.230–0.983), respectively. The occurrence of Cryptosporidium infection and associated risk factors identified through patient questionnaires are summarized in Table 2.

While the prevalence of *Cryptosporidium* was higher among individuals residing in apartments (8.1%) compared to those living in houses (4.8%), this difference was not statistically significant. Among the three institutionalized individuals included in the study, none tested positive for *Cryptosporidium*. Of the 312 patients, 168 reported diarrhea within the past six months and *Cryptosporidium* infection was detected in 10. However, similar observation were noted in individuals who did not report diarrhea.

Most patients reported consuming potable water. Despite this, *Cryptosporidium* was identified in 13 (6.6%) of them. Some patients—both infected and non-infected—were uncertain about the potability of their water supply or whether it had been tested.

When asked about fruit-washing practices before consumption, most patients (224; 96.3%) responded affirmatively. *Cryptosporidium* was detected in both those who regularly washed their fruits and those who did so only occasionally or not at all. Most patients did not frequently visit swimming pools (79.5%), however *Cryptosporidium* was still detected in this group. Patients who visited children’s playgrounds exhibited a higher prevalence of *Cryptosporidium* infection. Questionnaire responses indicated that few patients used public transportation (13.8%), however *Cryptosporidium* infection was still detected among those who did. Only a small number of study participants had traveled abroad. *Cryptosporidium* infection was detected in both travelers and non-travelers. Among the surveyed patients, only six individuals (1.9%) reported prior knowledge of cryptosporidiosis. None of these individuals tested positive for the infection.

### 3.3. Risk Factors Related to Animal Contact

Although *Cryptosporidium* was more prevalent in patients who had contact with animals, the differences were not statistically significant (Table 3). Similarly, *Cryptosporidium* was more frequently detected in pet owners than in non-pet owners, but this difference was also not statistically significant. No significant variation in prevalence was observed between owners of different animal species. Positive cases of *Cryptosporidium* infection were identified among owners of the following animal categories: cats, dogs, cats and dogs, or dogs and birds.

Animal groups that were reported by only one tested patient are not included in Table 3, particularly because those individuals tested negative for *Cryptosporidium* infection.

### 3.4. Handwashing Practices

Most patients reported washing their hands regularly after contact with animals. Nevertheless, 6% of individuals who reported practicing hand hygiene tested positive for Cryptosporidium infection. Patients who admitted to not washing their hands after animal contact had the highest positivity rate (11.1%). However, no statistically significant difference was observed when compared to participants who practiced handwashing.

We conducted a multivariate regression, binary logistic, model to identify independent risk factors for testing positive. No age group or sex significantly influenced the probability of being positive for *Cryptosporidium* in this sample. The 11–17 age group was virtually excluded from the analysis (0 positive cases). Multivariate logistic regression analysis was also ran for Table 2 (environmental and behavioral factors). All coefficients are insignificant (*p* > 0.5), and confidence intervals are wide. In this situation a simplified and stabilized version of the logistic regression analysis was performed by recoding all variables into binaries. No variable is significantly associated with a positive *Cryptosporidium* result, even after simplification. The situation was similar for Table 3.

Table 4 presents the primary diseases for which the patients were admitted to the infectious diseases departments. It can also be observed that *Cryptosporidium* infections were identified not only in patients with diarrhea.

## 4. Discussion

Little information on human cryptosporidiosis in Romania is available to the international medical community. This study represents the first investigation of Cryptosporidium infection risk factors conducted in western Romania. Our survey analyzed several risk factors in hospitalized patients from two departments of the Infectious Diseases Hospital in Timișoara. The overall prevalence was 5.77%; this may be attributed to the presence of various underlying pathologies among the study patients, which could serve as potential risk factors, increasing their susceptibility to *Cryptosporidium* infection. The complete agreement between the CerTest Crypto assay and the Ziehl–Neelsen staining method supports the reliability of the diagnostic approach used in this study. A study carried out in Ethiopia found that 11.5% of hospitalized patients were affected by cryptosporidiosis [19]. On a global scale, the infection rate is estimated at approximately 7.6%, with prevalence ranging from 4.3% in high-income nations to 10.4% in low- and middle-income countries [7]. An extensive and multifaceted investigation into cryptosporidiosis prevalence across multiple Eastern European nations revealed the following differing outcomes: 0% in Croatia and Bosnia and Herzegovina, 0.03% in Hungary, 1.53% in Slovenia, 3.34% in Estonia, and 4.04% in Romania. In the Czech Republic, the prevalence among AIDS patients was 10.71%, while in Serbia cryptosporidiosis was detected in 10.5% of HIV-positive patients. The prevalence found in this study exceeds that reported in earlier European research and could be linked to the underlying health conditions of the patients included [5].

Although differences in *Cryptosporidium* prevalence among various age categories were not statistically significant, there were slight differences between different age groups. Higher infection rates were observed in children aged 1–5 years and in individuals aged 40–59 years. The lower prevalence among infants under one year old could be attributed to their predominantly breast milk-based diet. In developed countries, research shows that cryptosporidiosis affects individuals of all ages regardless of their immune status [20], while in less developed regions, the infection is more commonly found among children and people living with HIV. In children, *Cryptosporidium* infections are typically identified after the age of 2 in developed countries and before the age of 2 in developing and underdeveloped countries. The prevalence of infection was apparently higher in men than in women; however, the differences were not significant, indicating that gender cannot be considered a risk factor. Wang et al. also concluded that age and sex are not significantly associated with the *Cryptosporidium* infection [21].

In this study, the prevalence of *Cryptosporidium* infection was notably higher (*p* < 0.05) among patients from urban areas, which may be attributed to a wider range of infection sources. A recent global review also found significantly greater infection rates in urban settings (23%) compared to rural ones (12.5%) [14]. These differences could be attributed to various factors, including higher population density, water quality, inadequate sanitation, and certain pathologies associated with cryptosporidiosis [11,12,13,14]. Contrary to these results, Yang et al. reported a higher prevalence of *Cryptosporidium* infection in rural areas of southwest China. In their study, a significant association was identified between hepatitis B virus (HBV) infection and *Cryptosporidium* infection [20].

Although the prevalence among patients living in apartments was almost double that of those living in houses, the differences were not statistically significant. It is possible that living conditions may have played a role in the *Cryptosporidium* infection. Similar results have been reported by other authors [21]. While some studies have demonstrated a higher prevalence of infection among patients with diarrhea, in our study, the prevalence differences between patients with and without diarrhea were not statistically significant [15,21]. The prevalence among patients with diarrhea (6%) was similar to that of patients without diarrhea (5.6%), both close to the overall prevalence (5.77%) of *Cryptosporidium* infection. The absence of significant differences is challenging to interpret, especially considering that cryptosporidiosis does not always present with diarrhea, and many patients in this study were receiving antibiotic treatment [15,22,23].

Several studies in the scientific literature suggests that water can be a source of the *Cryptosporidium* infection [24,25,26]. In the present study, *Cryptosporidium* infection was identified in patients who either did not know whether their drinking water was potable, did not know if the water had been tested, or had consumed tested potable water. However, the differences among these three categories were not statistically significant.

A study on cryptosporidiosis prevalence revealed that it is influenced by various risk factors, including international travel, human contact, swimming, poor hygiene, and overcrowding [9]. However, in our study, some of these potential risk factors, such as washing fruits, attending swimming pools, using public transport, and visiting children’s playgrounds, did not show statistical significance. Likewise, international travel did not influence infection prevalence. Nevertheless, when summing the responses “yes” and “sometimes” regarding these risk factors, a higher percentage of infection positivity was observed among patients exposed to these factors.

Furthermore, while our study observed that infection with *Cryptosporidium* occurred only among patients who lacked prior knowledge of the disease, this association alone is not sufficient to establish a preventive role for awareness. However, previous studies have suggested that increased public knowledge and awareness may contribute to improved hygiene practices and reduced risk of transmission, particularly in high-risk populations [27].

Patients who had contact with animals showed a marginally higher prevalence of cryptosporidiosis, but this difference lacked statistical significance. Similarly, animal owners had a higher prevalence of infection, but it was not statistically significant. When analyzed separately, different animal groups among pet owners did not significantly influence the prevalence; however, higher infection rates were observed in patients who own cats and dogs. This suggests that these animal species could represent potential risk factors, a notion also supported by other authors [28].

Studies conducted previously have identified that materials associated with farming activities, such as feces and manure from domestic animals, can be important contributors to the risk of cryptosporidiosis [14,20]. Handwashing, as a fundamental hygiene practice, is a preventive measure against *Cryptosporidium* infection [25]. Nevertheless, some patients reported not washing their hands after contact with animals. This could explain why the highest infection prevalence (11.1%) was found in this category. However, the differences between those who adhere to hand hygiene and those who do not were not statistically significant.

Unlike the current study, research conducted on children in Gambia found a link between *Cryptosporidium* infection and the consumption of stored drinking water, along with contact with certain animals such as cattle, cats, and rodents [15]. Patients who tested positive for the *Cryptosporidium* infection presented with various comorbidities. The majority of those with diarrhea were diagnosed with enterocolitis, particularly due to *C. difficile* and rotaviruses. While patients with diarrhea could prompt a clinical suspicion of cryptosporidiosis, asymptomatic individuals may represent less easily identifiable sources of infection within hospital patient communities. Therefore, stool examinations would be advisable prior to hospital admission.

## 5. Conclusions

*Cryptosporidium* spp. was identified in hospitalized patients from western Romania with and without diarrhea, with an overall prevalence of 5.77%. The infection was more common in urban areas (9.2%), the only statistically significant risk factor. These findings highlight the need for further investigation into urban-specific transmission factors. Further research should include a larger patient sample to assess the potential involvement of additional risk factors.

Asymptomatic carriers of *Cryptosporidium*, often overlooked due to the absence of diarrhea, may pose a hidden risk of transmission in hospital settings, highlighting the importance of routine stool screening before admission.

## Figures and Tables

**Table 1:** Distribution by age and gender of positive cases with *Cryptosporidium* spp.

Variable		Number of Tested Subjects n = 312	Number of Patients with Positive Test Results ^3^ n = 18 (%)	OR ^1^ 95%CI ^2^	*p*-Value
**Age group** **(years)**	<1	32	1 (3.1)	Ref.	
	1–5	64	5 (7.8)	2.62 (0.29–23.49)	0.66
	6–10	18	1 (5.6)	1.82 (0.1–31.03)	1
	11–17	4	0 (0)	-	1
	18–39	38	2 (5.3)	1.72 (0.15–19.9)	1
	40–59	43	3 (7)	2.33 (0.23–23.45)	0.63
	≥60	113	6 (5.3)	1.74 (0.2–15)	1
**Gender**	Female	166	8 (4.8)	Ref	
	Male	146	10 (6.8)	1.45 (0.56–3.78)	0.47

Legend: ^1^ Odds ratio; ^2^ confidence interval; ^3^ the results were identical using both methods.

**Table 2:** Occurrence and risk factors linked to human infection with *Cryptosporidium* spp. from Romania ascertained by questionnaire.

Variables		Number of Tested Subjects n = 312	Number of Patients with Positive Test Results ^4^ n = 18 (%)	OR ^1^ 95%CI ^2^	*p*-Value
**Area of residence**	Rural	193	7 (3.6)	2.7 (1.02–7.19)	**0.05**
	Urban	119	11 (9.2)		
**Housing**	House	210	10 (4.8)	Ref	
	Apartment	99	8 (8.1)	1.76 (0.67–4.6)	0.3
	Institutionalized	3	0	Undef ^3^	1
**Digestive symptoms in the last 6 months**	Yes	168	10 (6)	Ref	
	No	144	8 (5.6)	0.93 (0.36–2.42)	1
**Potable water source**	Yes	196	13 (6.6)	Ref	
	No	26	1 (3.8)	0.56 (0.07–4.49)	1
	Do not know	90	4 (4.4)	0.65 (0.21–2.07)	0.6
**Fruits washed before consumption**	Yes	224	14 (6.3)	Ref	
	No	18	1(5.6)	0.88 (0.11–7.12)	1
	Sometimes	70	3 (4.3)	0.75 (0.16–3.43)	1
**Frequent swimming in pools**	Yes	22	0	Ref	
	No	248	16 (6.5)	Undef ^3^	0.38
	Sometimes	42	2 (4.8)	Undef ^3^	0.54
**Frequent visits to children’s playgrounds**	Yes	83	4 (4.8)	Ref	
	No	185	10 (5.4)	1.13 (0.34–3.7)	1
	Sometimes	44	4 (9.1)	1.98 (0.47–8.31)	0.45
**Using public transport**	Yes	43	0	Ref	
	No	206	13 (6.3)	Undef ^3^	0.13
	Sometimes	63	5 (7.9)	Undef ^3^	0.08
**Frequent travelling abroad**	Yes	26	1 (3.8)	Ref	
	No	270	16 (5.9)	1.57 (0.2–12.38)	1
	Sometimes	16	1 (6.3)	1.67 (0.1–28.7)	1
**Have you heard of cryptosporidiosis?**	Yes	6	0	Ref	
	No	306	18 (5.9)	Undef ^3^	1

Legend: ^1^ Odds ratio; ^2^ confidence interval, ^3^ undefined; ^4^ the results were identical using both methods.

**Table 3:** Occurrence and risk factors linked to human infection with *Cryptosporidium* spp. from Romania related to contact with animals.

Variables		Number of Tested Subjects n = 312	Number of Patients with Positive Test Results ^4^ n = 18 (%)	OR ^1^ 95%CI ^2^	*p*-Value
**Contact with animals**	Yes	133	8 (6)	0.92 (0.35–2.41)	1
	No	179	10 (5.6)		
Having the animals in the household *	Yes	123	8 (6.5)	0.8 (0.3–2.1)	0.8
	No	189	10 (5.3)		
**Species of household animals**	With animals	123	8 (6.5)	Ref	
	Cats	13	2 (15.4)	0.38 (0.07–2.02)	0.24
	Dogs	49	4 (8.2)	0.78 (0.22–2.72)	0.74
	Dogs and cats	29	1 (3.4)	1.94 (0.23–16.22)	1
	Dogs and cats and birds	7	0	Undef	1
	Dogs and birds	2	1 (50)	0.06 (0.00–1.21)	0.11
	Birds and sheep	4	0	Undef ^3^	1
	Pigs and birds	2	0	Undef ^3^	1
	Birds	4	0	Undef ^3^	1
**Washing hands after contact with animals**	Yes	217	13 (6)	Ref	
	Sometimes	64	3 (4.7)	0.77 (0.21–2.8)	1
	No	18	2 (11.1)	1.96 (0.41–9.46)	0.32
	No answer	13	0	Undef ^3^	1

Legend: ^1^ Odds ratio; ^2^ confidence interval; ^3^ undefined; ^4^ the results were identical using both methods; * animal groups owned by only a single tested patient were not included in the table, especially since those patients were not positive for *Cryptosporidium* infection.

**Table 4:** Description of cases testing positive for *Cryptosporidium* spp.

No. Crt	Diagnosis	Diarrhea	Sex	Age (Years)	Rural (R)/Urban (U)
**1.**	Enterocolitis with C. difficile *, HIV, TBC	Yes	M	36	R
**2.**	Enterocolitis with C. difficile *, HTN	Yes	M	79	R
**3.**	Enterocolitis with C. difficile *	Yes	M	47	U
**4.**	Enterocolitis with C. difficile *	Yes	F	2	R
**5.**	Enterocolitis with C. difficile *, Type 2 Diabetes Mellitus	Yes	F	71	R
**6.**	Enterocolitis with C. difficile *, Heart failure, Morbid obesity	Yes	F	73	U
**7.**	Enterocolitis with C. difficile *, HTN	Yes	F	81	U
**8.**	Rotavirus enterocolitis	Yes	M	1	U
**9.**	Measles	Yes	F	1	R
**10.**	Measles	Yes	M	10 months	R
**11.**	Measles	No	F	25	U
**12.**	HIV	No	M	55	U
**13.**	Febrile syndrome	No	M	9	U
**14.**	Febrile syndrome	No	F	1	U
**15.**	Febrile syndrome, Hepatocytolysis syndrome	No	M	58	U
**16.**	Febrile syndrome	No	M	78	U
**17.**	Convulsive cough	No	F	3	R
**18.**	COVID-19, Parkinson’s disease	No	M	75	U
**Total**		10 Yes	10M + 8F	10 months–81 years old	7R + 11U

Legend: * Treated with Vancomycin; HIV—human immunodeficiency virus; HTN—hypertension; TBC—tuberculosis.

## Data Availability

The original contributions presented in the study are included in the article, further inquiries can be directed to the corresponding authors.
